# A tiered spatial validation workflow for geographic prediction models

**DOI:** 10.1016/j.mex.2026.104072

**Published:** 2026-07-25

**Authors:** Xueqian Jiang, Jialun Wu, Jiabei Liu

**Affiliations:** a Independent Researcher, Chaoyang, Beijing, 100004, China; b Independent Researcher, Jersey City, NJ, 07306, USA; c Northeastern University, 360 Huntington Ave, Boston, MA, 02115, USA

**Keywords:** Spatial validation, Cross validation, Geographic transfer, Graph partition, Spatial autocorrelation, Model evaluation

## Abstract

Geographically indexed observations violate the exchangeability assumed by ordinary random validation. We present a tiered spatial validation workflow that separates interpolation within an observed geographic mixture from transfer to a distinct area. The workflow combines random folds, a size matched random control, coordinate clusters, spectral communities of a nearest neighbor graph, administrative holdouts, and an external regional stress test. It reports pooled R squared and root mean squared error, repeated seed variability, spatial autocorrelation, and sensitivity to block scale. The workflow is dataset agnostic and is demonstrated on 211 geolocated Baltimore house sales predicted from structural attributes. Random validation exceeded coordinate cluster and graph partition validation, and the size matched control ruled out a training size artifact. Administrative and external region holdouts reduced apparent performance further, and coordinate cluster validation was closer to true external region transfer than random validation but remained optimistic. The workflow is intended for geographic machine learning studies that need to match performance claims to a stated deployment setting.

•The workflow distinguishes interpolation, separation within an observed network, and external geographic transfer.

•Matched size controls, repeated partitions, autocorrelation diagnostics, and regional holdouts make the validation gap interpretable.

•A dataset agnostic reference implementation is demonstrated on a public housing dataset unrelated to the motivating application.

## Graphical abstract

**Figure:**
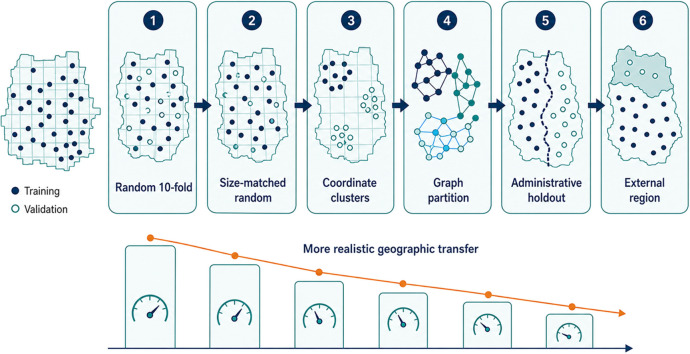

Submitted as a separate file named Graphical_Abstract.png.

## Background

### Specifications table

**Table:** 

Subject area	Computer Science
More specific subject area	Spatial machine learning and model evaluation
Name of your method	Tiered spatial validation workflow for geographic prediction models
Name and reference of original method	Cross validation for structured data [1], spatially separated folds [2], spectral clustering [3], and Moran's I [4]
Resource availability	The demonstration dataset loads from the PySAL project [5,6]. A dataset agnostic reference implementation will be deposited in a public GitHub repository upon acceptance.

Cross validation estimates how a model may perform on observations not used for fitting [7]. Random folds are appropriate when observations can reasonably be treated as exchangeable. Geographic data often violate this condition because nearby places share population composition, labor markets, health systems, policies, environmental exposures, and measurement processes. A random split can therefore place geographically similar or connected observations in both training and validation sets. The resulting score may measure interpolation within an observed geographic mixture rather than transfer to a new area [1,8].

Spatially separated folds are established in ecology, remote sensing, exposure modeling, and other fields [2 to 6]. No single blocking design is universally correct. A coordinate block, a graph community, a state, and an external region represent different deployment questions. Spatial blocking may also change training size, expose regional mean differences, or become unstable when blocks are too coarse [9,10]. A useful evaluation procedure must therefore compare several structured designs and explain what each estimate means.

Geographic units can be represented as nodes in a spatial network, with a spatial weights matrix serving as the network adjacency matrix [11,12]. Spatial autocorrelation quantifies similarity among connected units [4]. Coordinate clustering and graph partitioning can then create geographically separated folds using different representations of the same location information [3,13].

The workflow described here operationalizes these established ideas as a reproducible evaluation sequence for geographic machine learning. It does not claim that spatial cross validation, Moran's I, or spectral clustering is new. Its contribution is a tiered procedure that combines geometric folds, graph folds, training size controls, repeated seeds, model family checks, and an external transfer stress test. The workflow is demonstrated here on an independent public housing dataset [5,6], so that the procedure can be assessed apart from any single application.

## Method details

### Overview and required inputs

**Fig. 1:**
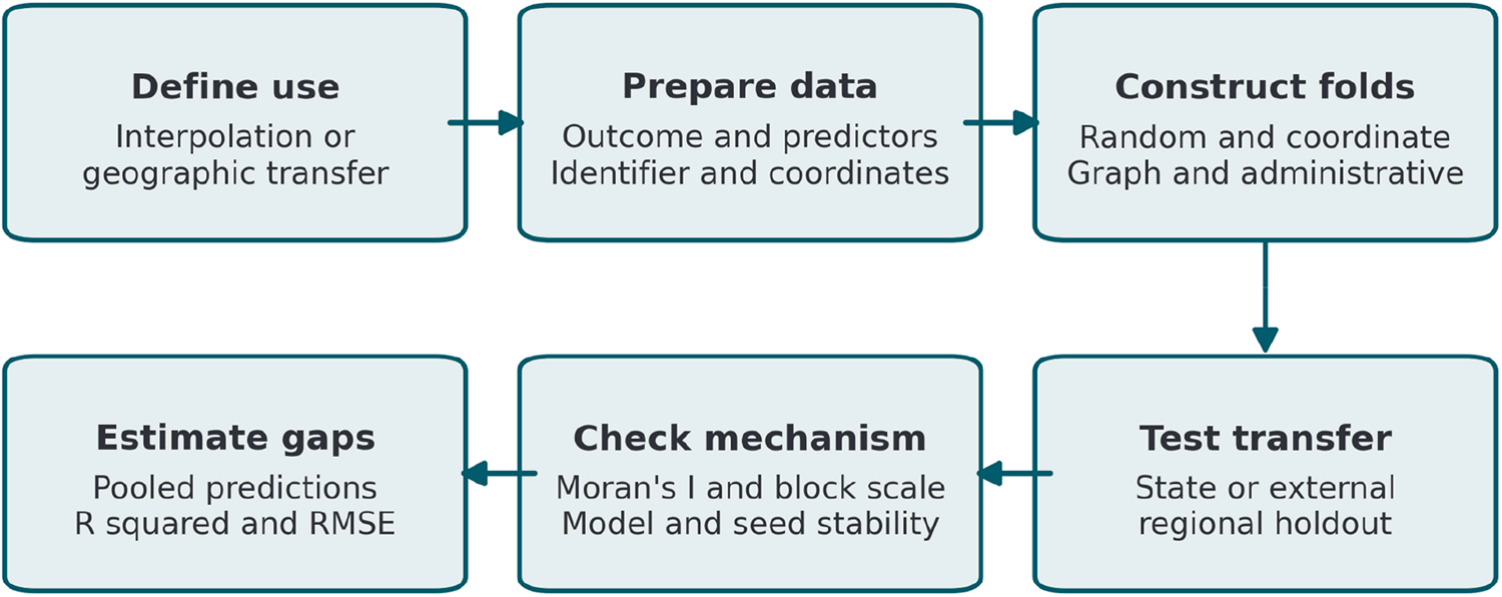
Tiered spatial validation workflow. The evaluation begins with the intended use and proceeds through data preparation, geographically structured folds, external transfer testing, mechanism checks, and gap estimation. The stages are interpreted together rather than as a ranking of interchangeable validation scores.

The workflow requires a tabular dataset with one row per geographic unit. Each row must contain a stable identifier, one outcome, predictor columns, and a representative longitude and latitude. Administrative group labels are required when state or jurisdiction holdouts are planned. An external region indicator is required when the intended claim concerns transfer beyond the observed development region. The same preprocessing pipeline and model specification must be fitted independently within every training fold. Fig. 1

### Data preparation and leakage controls

Merge outcome and predictor sources using the stable geographic identifier. Remove records with invalid coordinates and verify that each identifier occurs once. Define missingness thresholds before inspecting model performance. For each outcome, apply the same eligible unit mask to every validation scheme so that differences cannot be attributed to a changing evaluation sample. Imputation, scaling, feature selection, and hyperparameter selection must occur inside each training fold.

Predictors should be screened for direct or near direct versions of the target. Shared upstream data sources should be documented because they can strengthen predictor outcome relationships without constituting direct column leakage. The method evaluates geographic generalization and does not convert an associational model into a causal model.

The demonstration used the Baltimore house sales dataset distributed with the PySAL project [5,6]. Each of 211 geolocated sales carries a sale price, twelve structural attributes such as the number of rooms and bathrooms, lot size, interior area, and age, a city or county indicator, and planar map coordinates. The primary task predicted sale price from the twelve structural attributes. A panel of five continuous attributes was also predicted from the remaining attributes to exercise the cross outcome summaries. No records were missing, so the same 211 units entered every validation scheme.

### Validation designs

Begin with random ten fold cross validation as an interpolation benchmark. Create coordinate cluster folds by applying k means to the unit coordinates, using a fixed number of groups suited to the deployment scale, eight in the demonstration. Hold out each group once. Create a size matched random control whose test fold sizes match the coordinate cluster folds. This control isolates the effect of geographic grouping from the effect of changing the number of training and test observations.

Construct the neighbor graph from an eight nearest neighbor relation among unit coordinates. Symmetrize the relation so that an undirected edge is present when either unit selects the other as a neighbor. Remove self edges and verify the number of connected components. Apply spectral clustering to the adjacency matrix to create the same number of communities, then hold out each community once [3]. Coordinate clusters and graph communities both encode location by construction. Agreement between them is evidence of robustness to representation and partitioning objective, not evidence from independent geographic information.

Administrative holdouts group all units that share an administrative label, such as a state or jurisdiction, or the city and county indicator used in the demonstration. External region evaluation excludes the target region from all model fitting and internal validation, then evaluates the fitted model only in that region. The external region should be defined before outcome specific results are examined. Table 1 summarizes the intended interpretation of each scheme.

**Table 1:** Validation schemes and intended interpretation.

Validation scheme	Construction	Interpretation
**Random ten fold**	Units are assigned without geographic constraints.	Interpolation among units drawn from the same geographic mixture.
**Size matched random**	Random test folds match the sizes of coordinate cluster test folds.	Control for changes in training and test sample size.
**Coordinate cluster**	Unit coordinates are divided into k means groups and one group is held out at a time.	Transfer to a separated geometric block within the observed domain.
**Graph partition**	An eight nearest neighbor graph is divided into spectral communities and one community is held out at a time.	Transfer to a separated subgraph within the observed unit network.
**Administrative holdout**	All units sharing an administrative label are held out together.	Transfer across an administrative or jurisdictional boundary.
**External region**	A predefined region is excluded from all training and internal validation.	Transfer to a geographically distinct deployment region.

### Repeated model fitting

Repeat fold generation and model fitting across several fixed seeds. The demonstration used seeds 17, 42, 91, 123, and 202. Seeds varied random folds, k means initialization, spectral partition initialization, and model initialization where applicable. Report the mean score for each outcome and the standard deviation of the difference between validation schemes. Stable gaps across seeds indicate that the conclusion is not driven by one partition realization.

The primary model was ridge regression with median imputation, predictor standardization, and regularization chosen inside the training data over 13 logarithmically spaced values from 10 raised to minus 3 through 10 raised to 3 [14,15]. Random forest regression provided a nonlinear robustness check with 60 trees, a minimum leaf size of 5, and square root feature sampling [16]. The same predictors and unit masks were used across schemes within each model family.

### Performance measures and validation gaps

Pool held out predictions across all folds before calculating performance. Report R squared and root mean squared error, with root mean squared error kept in the original units of the outcome. Pooled R squared is intentionally sensitive to regional mean shifts because transfer to a new region requires prediction of its level as well as its internal ranking.

For a structured scheme s, define the validation gap as Δs = R²random − R²s. A positive value means that random validation reports better performance. Summarize the outcome distribution with the median, interquartile range, mean, and the number of positive gaps. The median and interquartile range are preferable primary summaries when a small number of outcomes have large gaps. A paired signed rank statistic can describe directional consistency, but outcomes measured in the same units are correlated and should not be treated as independent population units.

### Spatial autocorrelation and block scale

Calculate Moran's I for each outcome on the same spatial weights matrix used to define the neighbor graph [4]. Compare Moran's I with the validation gap across outcomes. A positive association supports the interpretation that stronger similarity among connected units produces greater optimism under random folds. This association remains descriptive because the panel outcomes are measured on the same units.

Repeat coordinate clustering across a range of block counts to assess scale sensitivity. More clusters create smaller held out areas and a task closer to local interpolation. Fewer clusters create larger geographic separation but reduce the training sample and increase fold variance. Interpret the average trend with its dispersion rather than requiring every outcome to change monotonically.

### Reference implementation

The reference implementation used Python with pandas, NumPy, SciPy, scikit learn, and Matplotlib [14]. It is provided as a dataset agnostic workflow module with a demonstration script and a figure script. The pipeline produces fold assignments, held out predictions, outcome summaries, graph partitions, and figures. Reproducibility requires fixed seed lists, explicit predictor lists, saved package versions, and a documented command order. The demonstration dataset loads directly from the PySAL project [5], so no external download is required. The analysis code will be deposited in a public GitHub repository upon acceptance.

## Method validation

### Demonstration on an independent housing dataset

**Fig. 2:**
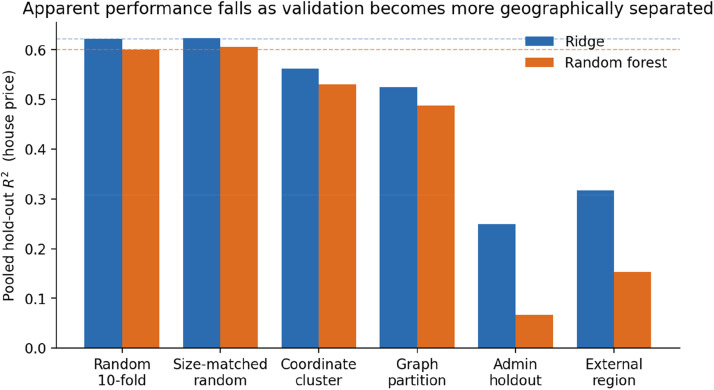
Pooled hold out R squared for house price by validation tier. Bars show mean performance over five seeds for ridge regression and random forest. Apparent performance falls as the validation split becomes more geographically separated. Dashed lines mark the random validation level for each model.

The workflow was demonstrated on 211 Baltimore house sales, predicting sale price from twelve structural attributes. Random ten fold validation was the most optimistic estimate. Mean R squared over five seeds was 0.622 under ridge regression and 0.601 under random forest. Apparent performance fell as folds became more geographically separated. For ridge regression it was 0.562 under coordinate cluster validation and 0.524 under graph partition validation; for random forest it was 0.531 and 0.488. The random minus coordinate cluster gap was 0.060 for ridge and 0.070 for random forest, and the random minus graph partition gap was 0.098 and 0.112. Seed to seed variability was small, with an R squared standard deviation of at most 0.016 across schemes. Fig. 2 shows the full ladder of estimates for both models.

### Model family, partition representation, and sample size

**Fig. 3:**
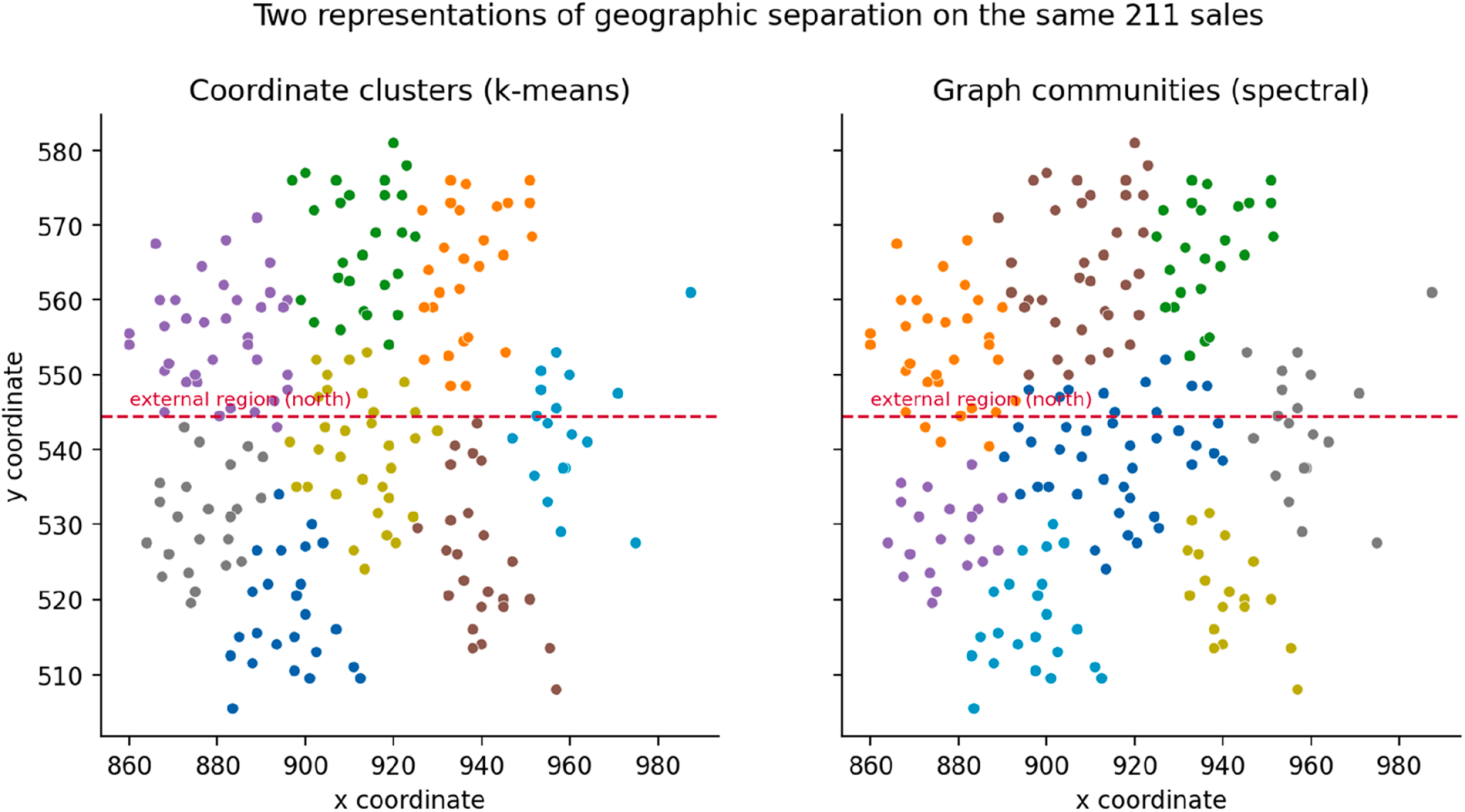
Two representations of geographic separation on the same 211 sales. Left, coordinate clusters from k means on the map coordinates. Right, spectral communities of the eight nearest neighbor graph. The dashed line marks the northern external region used for the transfer test.

Both model families reproduced the ordering. The neighbor graph contained 211 nodes, 990 undirected edges, a mean degree of 9.38, and one connected component. Coordinate clusters and graph communities partition the same coordinates, so their agreement is a robustness check across representations rather than independent evidence. Fig. 3 shows the two partitions together with the external region boundary.

The size matched random control was indistinguishable from ordinary random validation, with a mean gap of minus 0.002 and a maximum absolute gap of 0.014 across the panel. The drop under spatial blocking was therefore not an artifact of a smaller training sample. Administrative holdout across the Baltimore city and county boundary produced the largest reductions, with mean R squared of 0.249 for ridge and 0.067 for random forest, consistent with a genuine jurisdictional shift. Table 2 reports the primary task across all schemes.

**Table 2:** Validation evidence for the Baltimore house price task.

**Model**	**Validation scheme**	**Mean R squared**	**Gap versus random**
Ridge	Random ten fold	0.622	reference
Ridge	Size matched random	0.624	−0.002
Ridge	Coordinate cluster	0.562	0.060
Ridge	Graph partition	0.524	0.098
Ridge	Administrative holdout	0.249	0.373
Ridge	External region	0.317	0.305
Random forest	Random ten fold	0.601	reference
Random forest	Size matched random	0.605	−0.004
Random forest	Coordinate cluster	0.531	0.070
Random forest	Graph partition	0.488	0.112
Random forest	Administrative holdout	0.067	0.533
Random forest	External region	0.153	0.448

### Spatial autocorrelation and block scale

**Fig. 4:**
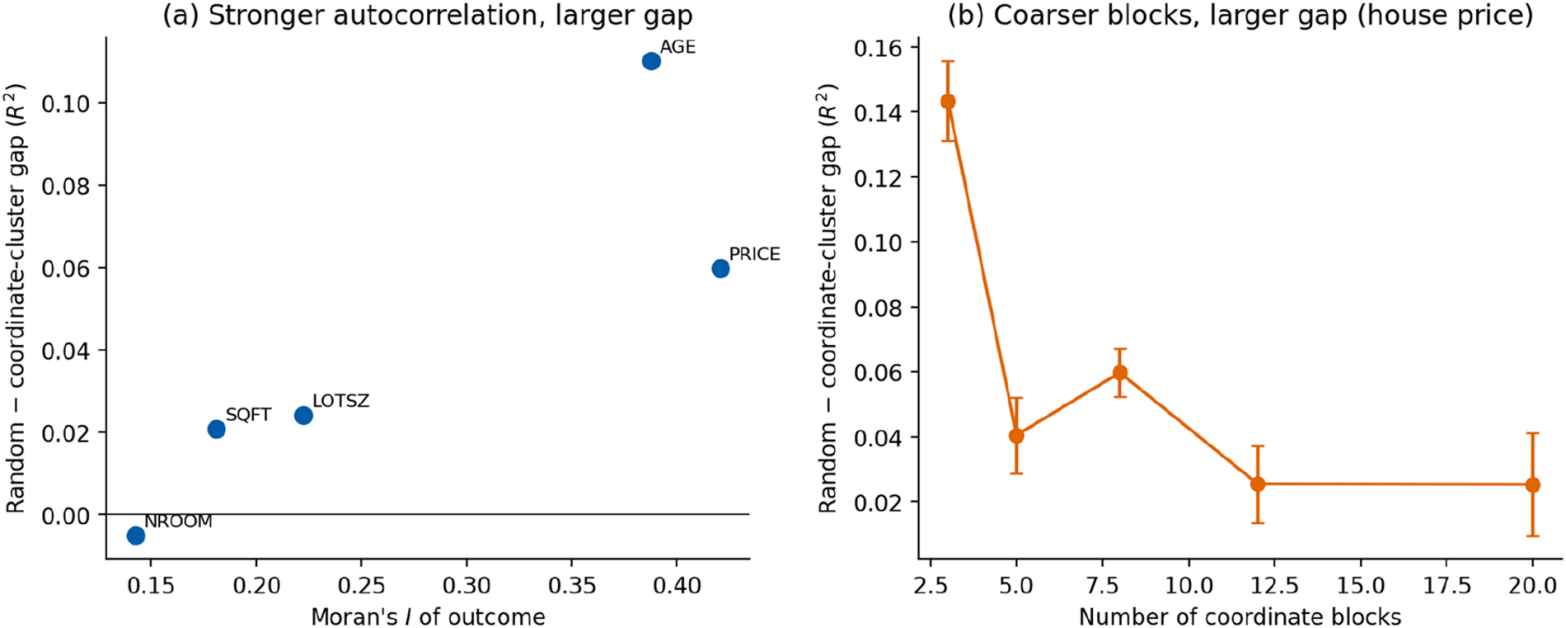
Diagnostics on the Baltimore demonstration. Panel a relates Moran's I of each panel outcome to its random minus coordinate cluster gap. Panel b shows the mean gap for house price as coordinate blocks become coarser, with bars marking one standard deviation across seeds.

A panel of five continuous attributes exercised the cross outcome summaries. Outcomes with stronger spatial autocorrelation showed larger gaps. Moran's I was 0.42 for sale price, 0.39 for age, 0.22 for lot size, 0.18 for interior area, and 0.14 for the number of rooms. The attribute with the weakest autocorrelation showed essentially no gap, while the most autocorrelated attributes showed the largest gaps, so the relationship is a positive trend rather than a strict ordering. The mean coordinate cluster gap for sale price increased as blocks became coarser, from 0.025 with twenty blocks to 0.143 with three blocks, and the coarsest partitions had greater dispersion because they reduced the training sample. Fig. 4 shows both relationships.

### External region stress test

The northern half of the study area was excluded from model development and then used as an external test set. Every internal estimate overstated northern transfer. For ridge regression the internal minus external difference in R squared was 0.305 for random validation, 0.245 for coordinate cluster validation, and 0.207 for graph partition validation; the random forest differences were larger. Coordinate cluster validation was closer to the external result than random validation in ninety percent of outcome and model cells, but remained optimistic. This reproduces on independent data the central conclusion that spatial validation reduces but does not remove transfer optimism [9,17,18].

The demonstration supports the intended use of the workflow. Random folds provide an interpolation benchmark. Coordinate clusters and graph communities test separation within the observed domain. Administrative and external region holdouts address progressively stronger transfer claims. Spatial validation is therefore necessary when geographic generalization is claimed, but an internal spatial split cannot guarantee performance in a genuinely new region.

### Limitations

The workflow does not identify one universally correct spatial split. Block geometry and scale must follow the deployment target, and coarse blocks may increase variance or reduce the training sample. Coordinate clusters and graph communities are derived from the same coordinates, so their agreement is a robustness check across representations rather than independent network evidence.

The demonstration dataset is small, with 211 sales in a single metropolitan area, so individual seed and partition realizations carry more variance than they would at larger scale, and the panel outcomes are measured on the same units. The demonstration is intended to show that the procedure behaves as designed on data unrelated to the motivating application, not to draw substantive conclusions about housing.

The external transfer test used one predefined region. Other settings may require state, service area, temporal, or institution based holdouts. Users should report the reason for their grouping and avoid treating a favorable internal spatial score as evidence for all possible deployment regions.

### Ethics statements

This work used only publicly available data. It involved no interaction with human participants and no access to identifiable private information. Ethics committee approval and informed consent were not required.

### CRediT author statement

Xueqian Jiang: Conceptualization, Methodology, Software, Formal analysis, Investigation, Data curation, Visualization, Writing – original draft.

Jialun Wu: Methodology, Validation, Formal analysis, Writing – review and editing.

Jiabei Liu: Validation, Data curation, Writing – review and editing.

All authors read and approved the final manuscript.

## Declaration of interests

### Declaration of generative AI and AI assisted technologies in the manuscript preparation process

During the preparation of this work, the authors used OpenAI Codex to support manuscript organization and language editing. The authors reviewed and edited the content and take full responsibility for the content of the article.

## Declaration of interests

The authors declare no known competing financial interests or personal relationships that could have appeared to influence the work reported in this paper.

## Data Availability

Data will be made available on request.
